# Optimization of fermentation conditions and medium components for chrysomycin a production by *Streptomyces* sp. 891-B6

**DOI:** 10.1186/s12866-024-03258-9

**Published:** 2024-04-06

**Authors:** Zhe Hu, Qiangang Weng, Zhehui Cai, Huawei Zhang

**Affiliations:** 1 School of Pharmaceutical Sciences, Zhejiang University of Technology, Hangzhou, 310014 China; 2 Key Laboratory for Green Pharmaceutical Technologies and Related Equipment of Ministry of Education, Zhejiang University of Technology, Hangzhou, 310014 China; 3 Key Laboratory of Pharmaceutical Engineering of Zhejiang Province, Hangzhou, 310014 China

**Keywords:** Chrysomycin, Marine *Streptomyces*, Fermentation, Optimization, Single-factor experiment, Response surface methodology

## Abstract

**Background:**

Chrysomycin A (CA) is a promising antibiotic for treatment of Gram-positive bacterial infections and cancers. In order to enhance CA yield, optimization of fermentation conditions and medium components was carried out on strain *Streptomyces* sp. 891-B6, an UV-induced mutant with improved CA titer compared with its wide-type marine strain 891.

**Results:**

Using one-way experiment, the optimal fermentation conditions for CA production in 1-L shake flask were obtained as follows: 12 days of fermentation time, 5 days of seed age, 5% of inoculum volume ratio, 200 mL of loading volume and 6.5 of initial pH. By response surface methodology, the optimal medium components determined as glucose (39.283 g/L), corn starch (20.662 g/L), soybean meal (15.480 g/L) and CaCO_3_ (2.000 g/L).

**Conclusion:**

Validation tests showed that the maximum yield of CA reached 1601.9 ± 56.7 mg/L, which was a 60% increase compared to the initial yield (952.3 ± 53.2 mg/L). These results provided an important basis for scale-up production of CA by strain 891-B6.

**Supplementary Information:**

The online version contains supplementary material available at 10.1186/s12866-024-03258-9.

## Background

**Fig. 1:**
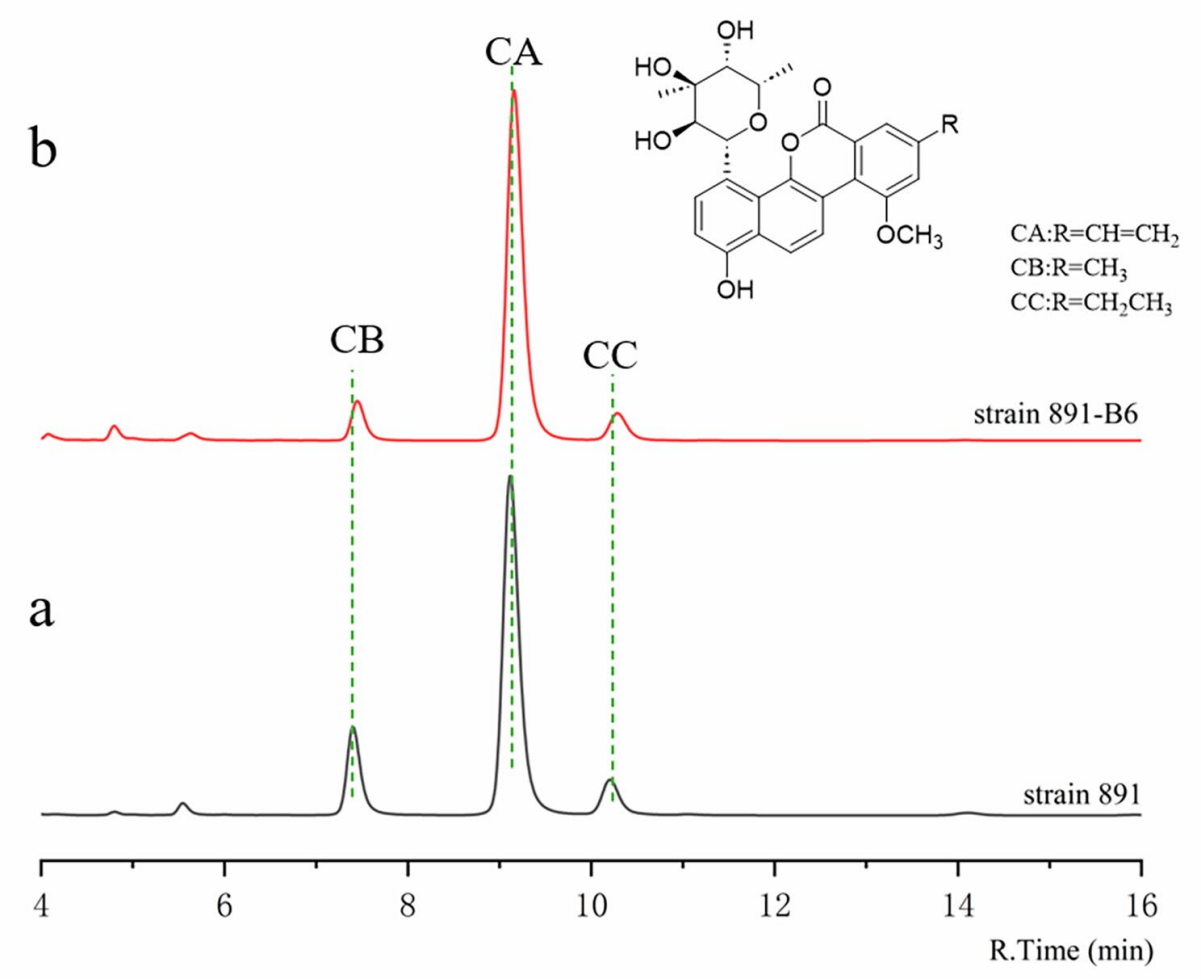
Comparison of HPLC profiles of fermentation extract of wide-type strain 891 (**a**) and UV-mutant strain 891-B6 (**b**)

Chrysomycins A-C (CA-CC, Fig. 1) are an unusual class of glycosides with a benzonaphthopyranone structure firstly discovered in 1955 from marine-derived strain *Streptomyces* sp. A-419 [1]. It had been demonstrated CA possesses remarkable antimicrobial activity against *Mycobacterium tuberculosis* (MT), multi-drug-resistant (MDR) tuberculosis and methicillin-resistant *Staphylococcus aureus* (MRSA) with MIC values of 3.125, 0.4, and 0.05 µg/mL, respectively [2–4], and also displays potent cytotoxic effect on human lymphoblastic leukemia HL-60, KRAS mutation cell NCl-H358 and glioblastoma U251 and U87- MG cell lines with IC_50_ values of 0.9, 0.15 0.475 and 1.77 µM, respectively [5–9]. Therefore, CA has the therapeutic potential for treatment of Gram-positive bacterial infections and cancers.

Strain *Streptomyces* sp. 891 originally from marine sediments had been shown to produce CA-CC with the ratio of 74:22:4 (Fig. 1a) [10]. Although the CA yield of strain 891 had been increased to 3648 ± 119 mg/L using single-factor and orthogonal experiments at 250-mL flask level, the CA content was invariable, causing the high cost of purification process [11]. Strain 891-B6 was obtained as one UV-induced mutant with higher CA content (89%) than that (74%) of the original strain (Fig. 1b) [12], suggesting this mutant is a more ideal strain for producing CA. In order to enhance CA production by strain 891-B6, this work highlighted optimization of fermentation conditions and medium components at 1-L flask level using one-way experiment and response surface methodology.

## Materials and methods

### Strain and medium

Strain 891-B6 was an UV mutant of the wild-type strain of *Streptomyces* sp. 891 and was stored at China General Microbiological Culture Collection Center (CGMCC No.21,775) [13]. ISP-2 was used as basic medium for cultivating strain 891-B6, which consisted of glucose 4 g/L, yeast extract 4 g/L, malt extract 10 g/L and agar 20 g/L.

### Fermentation condition

The seed of strain 891-B6 was prepared using ISP-2 medium and cultivated for 96 h at 30 ℃. Before fermentation, an aliquot of 10 mL seed solution was added to each 1-L flask with 200 mL fermentation medium containing corn starch (5.0 g/L), glucose (20 g/L), soybean meal (10 g/L) and CaCO_3_ (2.0 g/L). And the initial pH (7.0) was unmodified. The fermentation for CA production was carried out at 30 ℃ and 220 rpm in a shaker (ZS-AR, Zhejiang, China) for 10 d.

### Determination of CA yield

By the end of fermentation, 200 mL broth was centrifuged at 4000 rpm (TD5K, Changsha, China) for 20 min and the supernatant was removed. Mycelia of strain 891-B6 was extracted with 800 mL methanol using an ultrasonic extractor (G-080 S, Shenzhen, China) for 20 min at room temperature followed by filtration. An aliquot of 1 mL filtrate was further filtered using organic membrane with diameter of 0.22 μm for HPLC analysis.

### Structure identification of CA

Chemical structure of CA was unambiguously determined by a combination of various spectroscopic methods including H^1^- and C^13^-NMR and ESI-MS as well as comparison with literature data (see supporting material Figs. 1, 2 and 3; Table 1) [5].

### Single-factor experiment

Factors of fermentation condition for CA production were respectively evaluated at various levels, including, seed age from 4 to 9 d, inoculum volume from 2 to 10%, loading volume from 80 to 240 mL, initial pH from 6 to 8.5, glucose concentration from 10 to 50 g/L, corn starch concentration from 10 to 50 g/L and soybean meal concentration from 5 to 45 g/L.

### Response surface methodology for optimization of medium compositions

Based on the results of the above single-factor experiment, glucose, corn starch and soybean meal concentrations exhibited remarkable effect on CA yield. Therefore, these medium compositions were further optimized for CA production using response surface methodology based on Box-Behnken design (Design Expert 13.0, Stat-Ease Inc., Minneapolis, USA).

### Statistical analysis

Design Expert (version 13.0, Stat-Ease Inc., Minneapolis, USA) was used for analysis of variance (ANOVA) of Box-Behnken design. Each value was expressed as “mean ± standard deviation (SD)”. All experiments were performed three times in parallel.

## Results

### Effect of seed age on CA yield

**Fig. 2:**
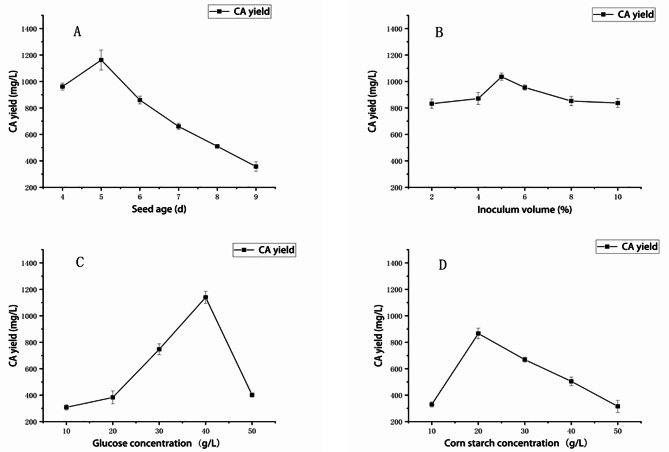
Effects of fermentation conditions on CA yield of strain 891-B6. (**A**: seed age; **B**: inoculum volume; **C**: glucose concentration; **D**: corn starch concentration)

Seed age is one of important factors affecting product yield since younger or older strain seeds lack strong vitality in their growth and metabolism. When the seed age is short, the formation of mycelial pellets is not conducive to the production of secondary metabolites [14, 15]. The experimental results showed that the highest CA yield (1162.7 ± 75.11 mg/L) achieved at 5-day, and the CA yield decreased significantly owing to mycelial aging (Fig. 2A). Thus, the optimal seed age for CA production is 5-day.

### Effect of inoculum volume on CA Yield

Inoculum amount of strain has an important impact on fermentation process since fewer inoculating volume usually slows down microbial growth and prolongs fermentation time and excessive volume frequently inhibits metabolic level [16]. As shown in Fig. 2B, the CA yield increased in a dependent manner within 2 to 5% of inoculum volume, which the best CA yield was 1035.9 ± 27.34 mg/L.

### Effect of glucose concentration on CA yield

Glucose is one of instant carbon sources for microbial growth and metabolism and its level in broth affects fermentation efficiency [17]. As shown in Fig. 2C, the best CA yield (1139.6 ± 45.6 mg/L) achieved when glucose concentration was 40 g/L. However, it rapidly decreased with the increasing concentration of glucose in fermentation medium.

### Effect of corn starch concentration on CA yield

Starch as macromolecular carbohydrate provides certain nutrients in the later stages of microbial fermentation, and its appropriate concentration in medium is conducive to biosynthesize secondary metabolites [18]. As shown in Fig. 2D, CA yield reached the highest level (866.3 ± 40.0 mg/L) at corn starch concentration of 20 g/L. However, it gradually declined in a concentration dependent manner within 20 to 50 g/L of starch concentration.

### Effect of soybean meal concentration on CA yield

**Fig. 3:**
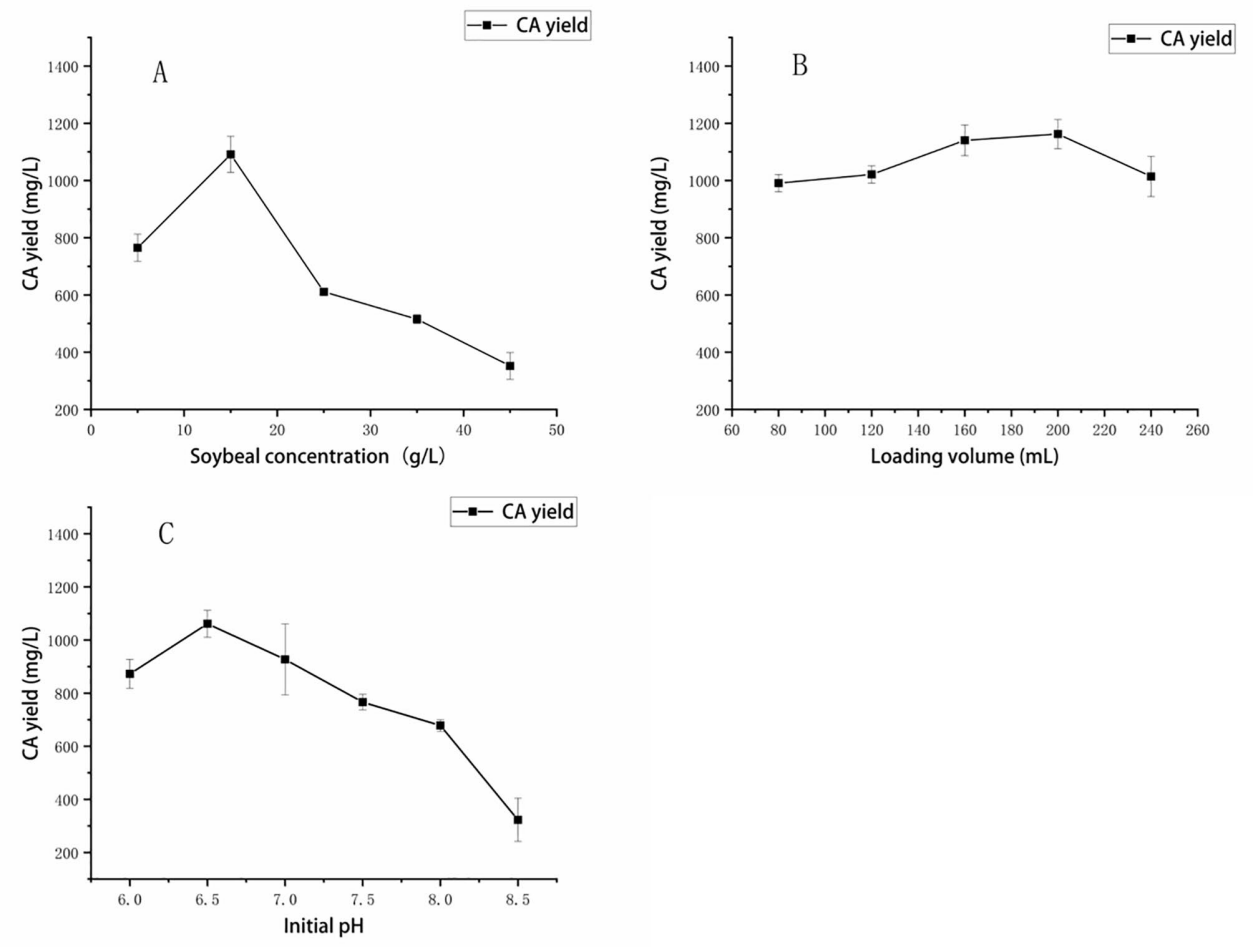
Effects of fermentation conditions on CA yield of strain 891-B6. (**A**: soybean meal concentration; **B**: loading volume; **C**: initial pH)

Soybean meal serves as an important nitrogen source for microbial growth and metabolism. It had been found that excessive nitrogen sources reduce CA yields of the wild strain 891 [19]. As shown in Fig. 3A, the CA yield reached up to 1091.3 ± 63.1 mg/L at the soybean meal concentration of 15 g/L and remarkably decreased as the concentration was higher than 15 g/L.

### Effect of loading volume on CA yield

Various loading volume in a fixed container affects microbial growth and metabolism, and appropriate liquid amount can ensure the demand of oxygen for strains during aerobic fermentation [20]. As shown in Fig. 3B, the highest yield of CA (1162.5 ± 54.18 mg/L) achieved when the loading volume was 200 mL in 1-L flask.

### Effect of initial pH on CA yield

It is well accepted that the optimum pH range for the growth of *Streptomyces* strains is from 6 to 8 [21]. As shown in Fig. 3C, the best CA yield was 1061.3 ± 51.04 mg/L at the initial pH 6.5. But it gradually decreased as the initial pH increased. This is maybe due to the fact that higher pH is unfavourable for those enzymes involved in CA biosynthesis.

### Response surface methodology

By response surface methodology based on Box-Behnken design, a total of 17 combination experiments were conducted with various concentrations of glucose, corn starch and soybean meal (Table 1). The results were analyzed to afford the following quadratic multinomial regression equation:

$$\eqalign{Y & =1546.40-125.96A+30.49C-27.95BC\cr &-608.64{A}^{2}-345.91{B}^{2}-388.14{C}^{2}}$$

where Y, A, B and C respectively represent the predicted CA yield, glucose, corn starch, soybean meal.

The ANOVA results shown in Table 2 suggested the regression of the model is highly significant. On basis of the F-value and the magnitude with the *P*-value, it was concluded that the degree of impact on CA fermentation is in the following order from the largest to the smallest: A > C > B.

**Table 1:** Experimental design and results of Box-Behnken optimization experiment

Std	Run	Glucose (g/L)	Corn starch (g/L)	Soybean meal (g/L)	CA yield (mg/L)
15	1	40	20	15	1550.5 ± 92.13
9	2	40	10	5	724.7 ± 68.72
16	3	40	20	15	1548.2 ± 83.54
4	4	50	30	15	498.8 ± 42.75
11	5	40	10	25	853.7 ± 88.42
2	6	50	10	15	425.8 ± 34.52
1	7	30	10	15	715.9 ± 48.31
8	8	50	20	25	449.7 ± 22.14
5	9	30	20	5	645.6 ± 56.35
17	10	40	20	15	1549.2 ± 66.46
13	11	40	20	15	1545.2 ± 100.17
6	12	50	20	5	404.8 ± 34.12
7	13	30	20	25	698.4 ± 23.24
10	14	40	30	5	826.9 ± 38.21
12	15	40	30	25	844.1 ± 47.16
3	16	30	30	15	726.9 ± 56.38
14	17	40	20	15	1538.9 ± 34.29

**Table 2:** ANOVA for the fitted quadratic polynomial model

Source	Sum of Squares	df	Mean Square	F-value	*P*-value
Model	3,130,000	9	347,700	4949.17	< 0.0001
A-Glucose	126,900	1	126,900	1806.62	< 0.0001
B-Corn starch	3898.45	1	3898.45	55.49	0.0001
C-Soybean meal	7435.9	1	7435.9	105.83	< 0.0001
AB	961.00	1	961.00	13.68	0.0077
AC	15.6	1	15.6	0.2221	0.6518
BC	3124.81	1	3124.81	44.48	0.0003
A^2^	1,560,000	1	1,560,000	22199.74	< 0.0001
B^2^	503,800	1	503,800	7170.72	< 0.0001
C^2^	634,300	1	634,300	9028.21	< 0.0001
Residual	491.82	7	70.26		
Lack of Fit	406.24	3	135.41	6.33	0.0534
Pure Error	85.58	4	21.39		
Cor Total	3,130,000	16			

**Fig. 4:**
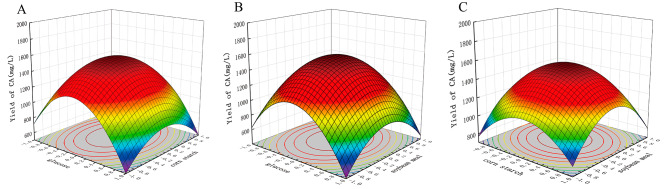
Response surface for CA production by strain 891-B6. (**A**: interaction between glucose and corn starch; **B**: interaction between glucose and soybean meal; **C**: interaction between corn starch and soybean meal.)

**Fig. 5:**
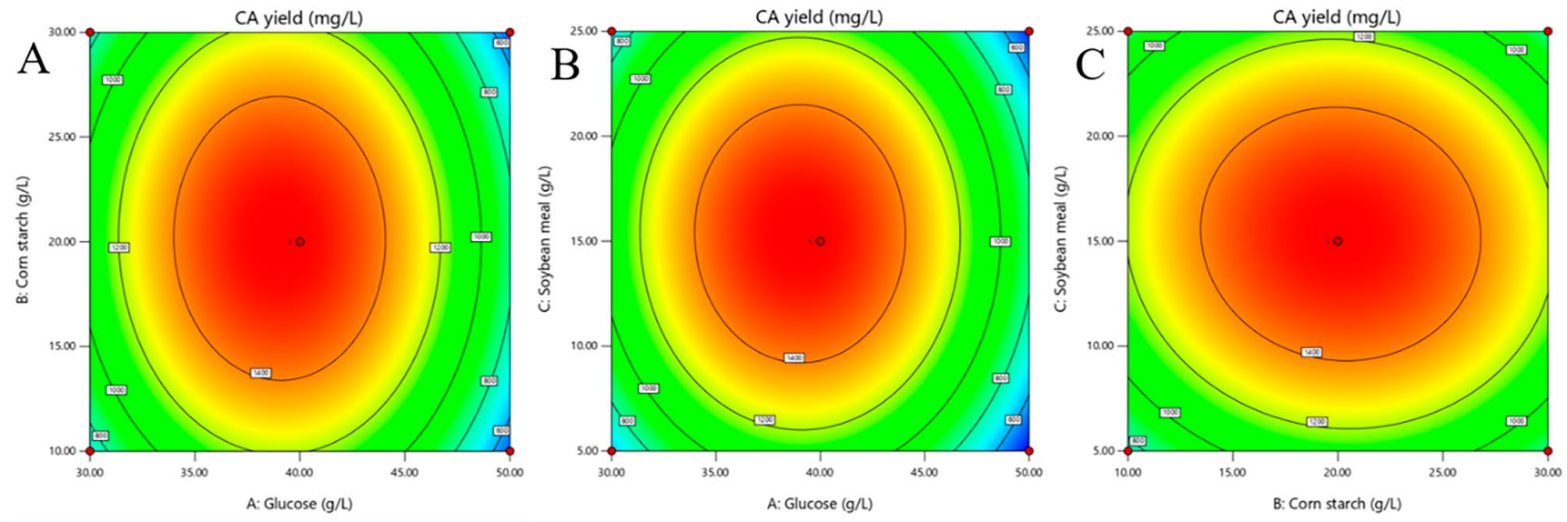
Two-dimensional contour map for factor interactions. (**A**: interaction between glucose and corn starch; **B**: interaction between glucose and soybean meal; **C**: interaction between corn starch and soybean meal.)

The effect of the optimum level of each variable and its interaction on CA yield was investigated by plotting three-dimensional response surfaces and two-dimensional contours for any two independent variables. Under the condition of glucose, corn starch and soybean meal with two certain factors, CA yield gradually increased at the beginning stage and reached the top level at nearby center of each factor, then gradually decreased with the increase of the third factor (Fig. 4). Both contour shapes of factor A (glucose) with factors B (corn starch) and C (soybean meal) were elliptical, indicating significant interactions, while the round contour shapes of factor B with C indicated a moderate interaction (Fig. 5). The model predicted a maximum CA yield of 1552.662 mg/L in 1-L flask when glucose, corn starch and soybean meal were 39.283, 20.662, 15.480 g/L, respectively.

### Verification result

**Fig. 6:**
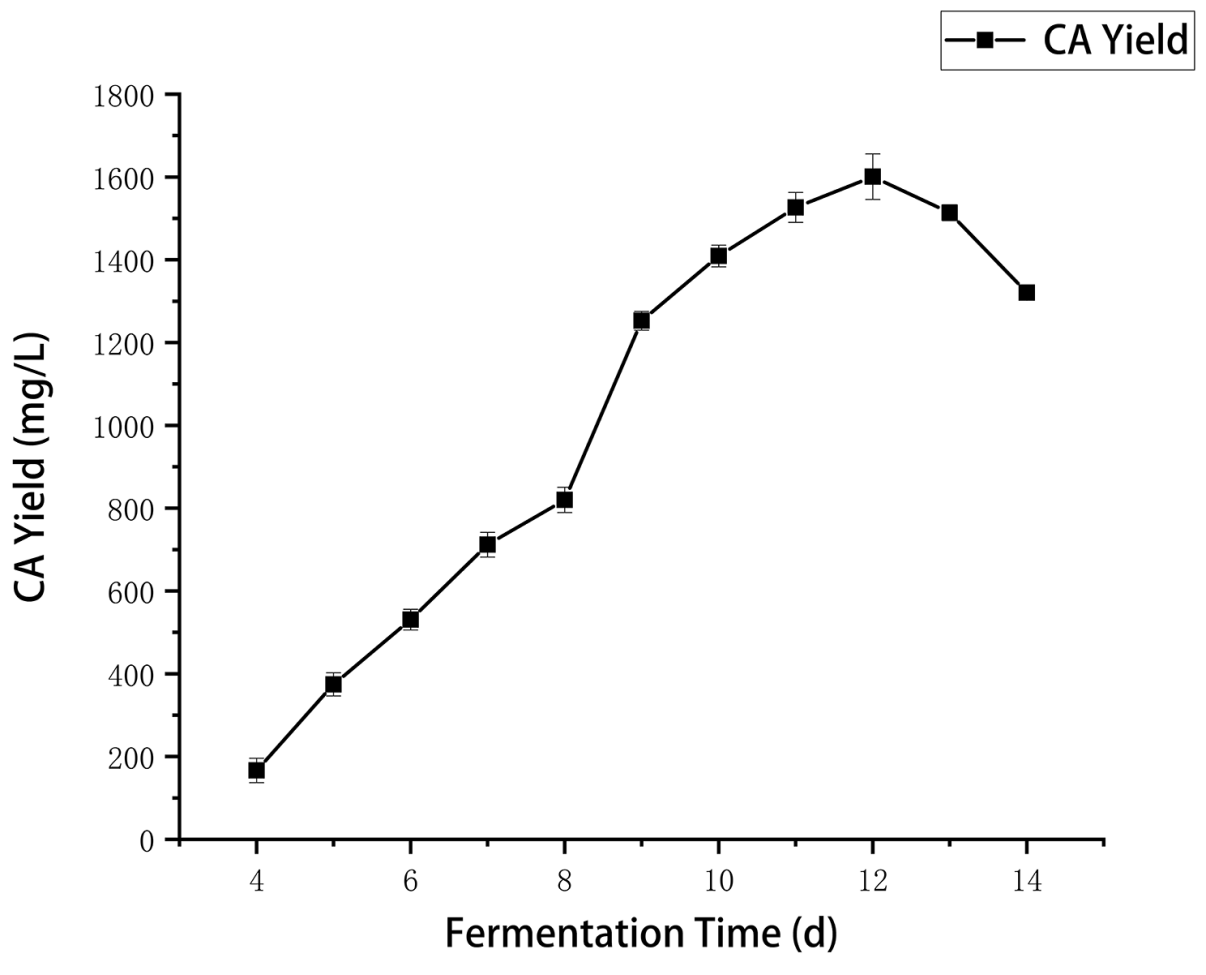
Effect of fermentation time on CA yield of strain 891-B6

At a seed age of 5 days, inoculum volume ratio of 5%, loading volume of 200 mL, initial pH 6.5, glucose 39.283 g/L, corn starch 20.662 g/L, soybean meal 15.480 g/L, CaCO3 2 g/L, the applicability of the model equations for predicting optimal response values was tested and fermentation time from 4 to 14 d was examined. As shown in Fig. 6, CA yield gradually increased in a fermentation time-dependent manner and reached the highest level (1601 ± 56.7 mg/L) at day 12, which was about 60% increase compared with the original titer (952.3 ± 53.2 mg/L) and showed good agreement with the predicted value (1552.662 mg/L). Therefore, the model developed in this study was adequate for reflecting the predicted optimization of CA production. As of day 12, however, the CA yield began to decrease probably due to the apoptosis of mycelia and/or CA breakdown [22]. So, the best fermentation time for CA production was determined as 12-day.

## Discussion

Antimicrobial resistance (AMR) has posed a global threat to humankind and could lead to annual deaths up to 10 million people by 2050 [23–26]. Vancomycin is one of the last-line antibacterial agents to treat MRSA infections for nearly four decades, and almost 20 years later several vancomycin-resistant *S. aureus* (VRSA) isolates had been discovered [27, 28]. However, clinical cases of VRSA (with MIC ≥ 16 µg/mL) and vancomycin-intermediate *S. aureus* (VISA) (with MIC > 8 µg/mL) are becoming increasingly common on earth [29]. Therefore, it is urgent to develop novel antibiotics with new actions of mechanism to combat AMR. CA as a drug lead has the great potential of therapeutic application owing to its potent bactericidal effect on MRSA by targeting multiple critical cellular processes [30]. In this study, optimization of fermentation conditions and medium components for CA production by the modified mutant 891-B6 at flask level were fulfilled by one-way experiments and response surface methodology. These results pave a foundational way for scale-up production of CA and would accelerate the development of new anti-AMR drugs.

The biosynthesis of CA in several wild strains is usually accompanied by the production of its analogs CB and CC, which pose a great challenge for large-scale production of pure CA. As we know, the increase of target product content effectively reduces its production cost [31, 32]. Therefore, the mutant 891-B6 with higher CA content is more suitable for industrial production of CA.

## Conclusion

By one-way experiments and response surface methodology, the optimal fermentation conditions and medium formulation for CA production by strain 891-B6 were determined as follows: 5 days of seed age, 5% of inoculum volume ratio, 200 mL of loading volume, 6.5 of initial pH, 39.283 g/L glucose, 20.662 g/L corn starch, 15.480 g/L soybean meal and 2 g/L CaCO_3_ and 12 days of fermentation time. Under these optimal conditions, the CA yield reached up to 1601.9 ± 56.7 mg/L, which was about 60% increase compared with the original level.

### Electronic supplementary material

Below is the link to the electronic supplementary material.

Supplementary Material 1

## Data Availability

No datasets were generated or analysed during the current study.

## Abbreviations

CA-CC: Chrysomycins A-C
MT: *Mycobacterium tuberculosis*
MDR: Multi-drug-resistant
MRSA: Methicillin-resistant *Staphylococcus aureus*
NMR: Nuclear Magnetic Resonance Spectroscopy
ESI-MS: Electrospray ionization mass spectrometry
SD: Standard deviation
AMR: Antimicrobial resistance
VRSA: Vancomycin-resistant *S. aureus*
VISA: Vancomycin-intermediate *S. aureus*
