# Supplementary material for: Optimization of fermentation conditions and medium components for chrysomycin a production by Streptomyces sp. 891-B6
Source: BMC Microbiol. 2024 Apr 6;24:120. doi: 10.1186/s12866-024-03258-9 (PMC10998411; doi:10.1186/s12866-024-03258-9)
Supplement: Supplementary file 1 — Supplementary Material 1 [file 12866_2024_3258_MOESM1_ESM.docx]

**Supporting Information**


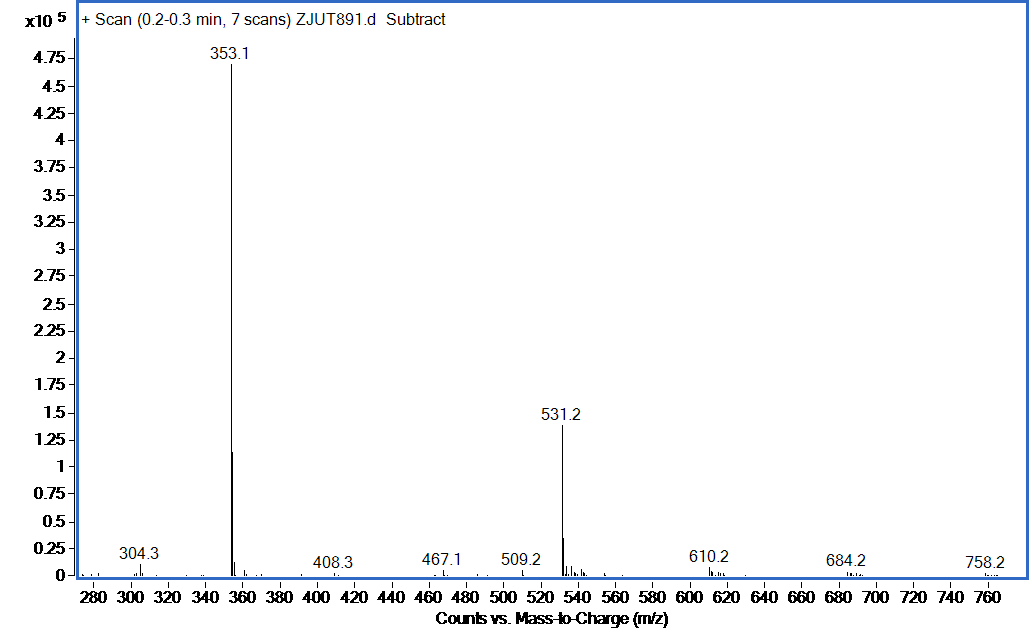


Fig. 1 The ESI-MS of CA

Fig. 2 The ^1^H NMR of CA

Fig. 3 The ^13^C NMR of CA

Table 1 Results of CA NMR

.

| Position | *δ*_H_ (*J* in Hz) | *δ*_C_ |
| --- | --- | --- |
| 1 |  | 153.2 |
| 1-OH | 9.80, s |  |
| 2 | 6.97, d (5.0) | 112.2 |
| 3 | 7.85, d (5.0) | 129.3 |
| 4 |  | 128.1 |
| 4a |  | 125.2 |
| 4b |  | 142.5 |
| 6 |  | 159.8 |
| 6a |  | 122.0 |
| 7 | 7.97, s | 119.1 |
| 8 |  | 138.8 |
| 9 | 7.68, s | 114.7 |
| 10 |  | 157.4 |
| 10a |  | 122.9 |
| 10b |  | 113.2 |
| 10-OCH_3_ | 4.14, s | 56.3 |
| 11 | 8.42, s | 101.5 |
| 12 |  | 151.8 |
| 12a |  | 115.2 |
| 12-OCH_3_ | 4.09, s | 56.7 |
| 13 | 6.92, dd (10.0, 15.0) | 135.2 |
| 14 | 6.13, d (15.0)  6.03, d (10.0) | 117.2 |
| 1’ | 5.49, d (5.0) | 74.7 |
| 2’ | 3.69, d (10.0) | 73.2 |
| 3’ |  | 72.6 |
| 4’ | 3.16, s | 75.9 |
| 5’ | 4.54, q (10.0) | 70.7 |
| 6’ | 1.26, s | 24.0 |
| 7’ | 1.03, d (10.0) | 17.1 |
